# Optimal Vaccination for Contagious Diseases with Seasonal Transmission

**DOI:** 10.1007/s11538-026-01741-0

**Published:** 2026-09-02

**Authors:** Nir Gavish, Guy Katriel

**Affiliations:** 1 Faculty of Mathematics, Technion Israel Institute of Technology, 32000 Haifa, Israel; 2 Department of Applied Mathematics, Braude College of Engineering, 2161002 Karmiel, Israel

**Keywords:** Mathematical Epidemiology, Seasonality, Vaccination, Optimal control

## Abstract

We study optimal periodic time-dependent vaccination strategies for seasonal epidemics with waning immunity, aiming to minimize the basic reproduction number under a fixed annual vaccine supply. Our analysis, which does not pre-assume the structure of the vaccination profile, reveals that the optimal time-periodic vaccination profile typically alternates between intervals of active, time-varying vaccination and intervals with no vaccination. Within the active periods, we derive an explicit expression for the optimal vaccination rate. For a wide class of continuous seasonal transmission rate functions, we show that the optimal solution does not exhibit pulse components. In contrast, when the transmission rate exhibits upward jump discontinuities, such as those arising from school-term forcing, the optimal strategy includes pulse components. These theoretical findings are supported by numerical computations of optimal vaccination profiles across a range of scenarios.

## Introduction

The ultimate aim of vaccination against a contagious disease is to eliminate the ability of a pathogen to spread in a population. Due to the principle of herd immunity, such elimination does not require vaccination of the entire population, but rather achieving a level of vaccination surpassing the herd immunity threshold, which depends on the contagiousness of the pathogen. In principle, if the herd immunity threshold is reached on a global level, and assuming there are no animal reservoirs, one can achieve eradication, which implies that further vaccination is not required (Martcheva 2015; Keeling and Rohani 2011). However, such global eradication has been achieved for only one human pathogen - the Smallpox virus, which was declared globally eradicated in 1980 (Breman et al. 1980). For other endemic pathogens, e.g., Measles, Rubella, Pertussis, and Polio, while local elimination in a given country is often achieved, there is an ever-present threat of re-introduction. In view of the continual renewal of the susceptible population, due to demographic turnover, waning of immunity, and microbial evolution, this implies that vaccination programs must be maintained indefinitely in order to prevent renewed spread.

Designing vaccination programs against endemic infectious diseases involves many issues, which may be fruitfully addressed with the aid of mathematical modeling. For example, a significant body of work uses multi-group models to study the optimal allocation of vaccines among different subpopulations of a heterogeneous population, e.g. age groups, occupational groups, or residents of different locations (Bai 2021; Ball et al. 2004; Duijzer et al. 2016; Hill and Longini 2003; Wallinga et al. 2010; Keeling and Shattock 2012; Kuddus et al. 2021; Medlock and Galvani 2009; Bansal et al. 2006). These efforts have naturally received widespread attention in the wake of the COVID-19 pandemic (Matrajt et al. 2020; Bubar et al. 2021; Islam et al. 2021; Moore et al. 2021; Gavish and Katriel 2022). Another line of research uses age-structured models to optimize age at vaccination, taking into account the effect of age on susceptibility to and risks of infections (Agur et al. 1993; Müller 1998, 2000). A further level of complexity arises from the fact that the outcomes of a vaccination campaign depend not only on health authorities’ vaccine allocation decisions, but also on individuals’ decisions regarding vaccine uptake. This motivates a range of approaches to modelling human behavior with respect to vaccination, coupled to epidemiological dynamics (Chang et al. 2020; Manfredi and D’Onofrio 2013; Scheckelhoff et al. 2021; Lopez et al. 2024).

This work explores a different dimension of the optimization of vaccination policies: We consider endemic diseases characterized by seasonal transmission and waning immunity, such as Influenza or COVID-19 (Kissler et al. 2020; Yaari et al. 2013; Truscott et al. 2012; Andreu-Vilarroig et al. 2025; Lofgren et al. 2007). A seasonal transmission pattern raises the prospect that vaccination strategies which vary in intensity according to season could be more effective than those which are constant in time, in the sense that they can achieve local elimination of the pathogen with lower vaccination effort. We show that this is indeed the case. Moreover, we pose and study the problem of finding an *optimal* time-periodic vaccination plan, achieving local long-term elimination with minimal effort. By local long-term elimination we mean ensuring that if the disease is reintroduced into the population at low levels, it will die out over time. Within our modeling framework, this requires finding the recurrent annual vaccination plan that stabilizes the disease-free equilibrium by reducing the reproductive number, $\mathcal {R}$, below one, using a minimal amount of vaccines per year.

A form of periodic vaccination which has been widely studied is ‘pulse vaccination’, in which a certain fraction of the population is vaccinated on a particular date every year (or every several years) (Agur et al. 1993; Altizer et al. 2006; Choisy et al. 2006; d’Onofrio 2002; Earn et al. 2000; Keeling et al. 2001; Khasin et al. 2010; Nokes and Swinton 1995; Rohani et al. 2000; Shulgin et al. 1998; Zhou and Liu 2003). Pulse vaccination can be considered the most extreme form of time-periodic vaccination, while the other extreme is vaccination at a constant rate (Liu and Stechlinski 2011). In Gabrick et al. (2024), numerical simulations are performed to compare vaccination at a constant rate with pulse vaccination, as well as with ‘pulse width’ profiles, which apply vaccination at a constant rate for part of the year. However, with an infinite-dimensional space of possible time-periodic schedules, there is no a priori reason to assume that pulse vaccination or any other pre-defined profile is optimal. The optimal vaccination profile may include pulse vaccination, continuous vaccination, or mixed strategies that combine continuous efforts with pulses and periods of inaction. Determining the optimal periodic vaccination profile leads to a nontrivial *optimal periodic control* problem.

To avoid imposing any *a priori* assumptions about profile structure or regularity, and to allow the optimal solution to include pulse vaccination, we need to allow our candidate profiles to be a class of *measures* rather than a class of bounded functions, as is commonly assumed in control theory. This leads us to an optimization problem over a space of positive periodic measures, whose analysis involves interesting technical challenges. We have performed a detailed analysis of this type of problem in Gavish and Katriel (2026), and here we apply some of the results of this previous work to the problem of optimal vaccination. Using these results, we will be able to obtain quite detailed information on the optimal vaccination profiles and their properties, by combining analytical results and numerical investigation. In particular, we will see that under certain circumstances the optimal vaccination profile includes pulse components - mathematically expressed as Dirac δ-functions. This fact shows that formulating the problem as an optimization problem over general measures rather than functions (which correspond to *absolutely continuous* measures), is essential - restricting the optimization problem to functions would lead to a problem without minimizers.

In mathematical epidemiology, control theory has been applied to the mitigation of epidemics, both using non-pharmaceutical measures and to problems in which vaccination is the control variable (Bliman et al. 2021; Choi and Shim 2021; Sharomi and Malik 2017; Lenhart and Workman 2007). However, these works optimize outcomes over a finite time horizon, and under assumptions that enable optimization over a space of functions, rather than of measures. In a different context, optimization over measures has been used to determine optimal vaccination ages (Müller 1998, 2000). To our knowledge, the only existing studies that, like the current study, aim to find an optimal periodic vaccination profile, with the aim of long-term elimination of the spread of a seasonal endemic disease, are due to Onyango and Müller (2014) and to Doutor et al. (2016). These works, which will be discussed in Sects. 2 and 9, treat problems which are closely related to the problem studied here, but with some crucial differences in the underlying assumptions, which lead to fundamental differences in the results.

The paper is organized as follows. In Sect. 2 we consider the problem of finding the optimal periodic vaccination profile and formulate it as an optimization problem over a space of measures. Section 3 is devoted to the characterization of the optimal vaccination profile, under some regularity conditions on the seasonal transmission profile β(t). The key results of this section are summarized in Theorem 4. This theorem shows that the optimal vaccination profile is an absolutely continuous measure and provides an explicit expression for its density on its support set. In Sect. 4 we consider the dependence of the optimal vaccination profile on the average vaccination rate $\bar{\eta }$. In particular, we analyze the limit of scarce vaccine supply  $\left. \bar{\eta }\ll 1\right.$, and the case of sufficiently large supply, and present a numerical study for intermediate values of $\bar{\eta }$. Section 6 considers the relative efficacy of the optimal vaccination profile compared to the use of a constant vaccination profile. In Sect. 7 we relax the regularity assumptions on the seasonal transmission profile β(t) and consider discontinuous transmission rates. The key result of this section is summarized in Theorem 10, which characterizes the behavior of the optimal vaccination profile at points of discontinuity of the transmission profile, in particular showing that upward jump points in the transmission rate lead to a pulse (Dirac δ-function) component in the optimal vaccination profile. In Sect. 8, we consider, using simulations, the behavior of the solution of the transmission model when the optimal vaccination profile is applied.

While the problem considered in this work - optimizing temporal vaccine allocation in a seasonal environment, with the aim of minimizing the reproductive number, is a very general one, our analysis of the problem is restricted by specific modelling assumptions. In section  9 we discuss several directions in which the basic model studied here can be extended. Investigating the same type of optimization problem in the context of more general models will be important in order to understand whether and how the nature of the optimal vaccination profile depends on the natural history of infection and decay of immunity, but also poses additional mathematical challenges. The current work is thus a step towards achieving a more general understanding.

## Statement of the Problem

Our analysis is based on a SIRVS (Susceptible-Infected-Recovered-Vaccinated-Susceptible) model that incorporates seasonality, vaccination, and waning immunity:

1 $$\begin{aligned} S^\prime= &  \mu (1-S)-\beta (t)SI+\delta _R R -\eta (t)S+\delta _V V,\nonumber \\ V^\prime= &  \eta (t)(S+R)-(\delta _V+\mu )V,\nonumber \\ I^\prime= &  \beta (t)SI -(\gamma +\mu )I,\nonumber \\ R^\prime= &  \gamma I-(\delta _R+\mu ) R-\eta (t)R. \end{aligned}$$

**Fig. 1:**
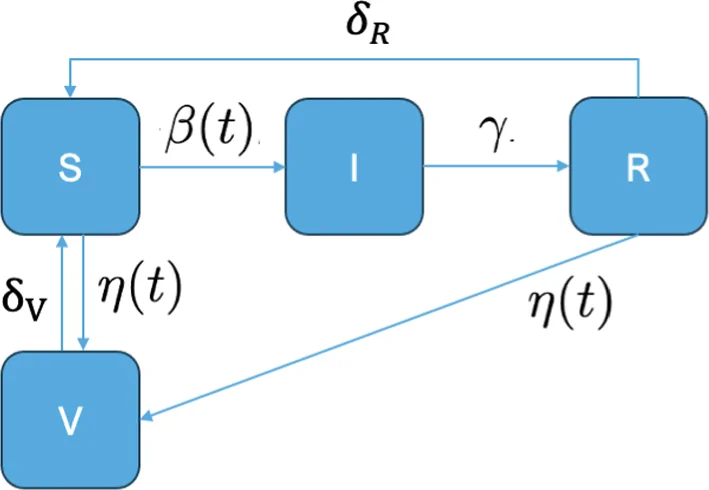
Flowchart diagram of the SIRVS epidemic model. The boxes represent the different population compartments: Susceptible (*S*), Infected (*I*), Recovered (*R*), and Vaccinated (*V*). The directed arrows indicate the transition pathways and their corresponding rates, including the time-dependent seasonal transmission rate β(t), recovery rate γ, waning of natural immunity $\delta _R$, waning of vaccine-induced immunity $\delta _V$, and the time-dependent vaccination rate η(t). Natural birth and death rates are omitted from this diagram for visual clarity

Here, *S*, *I*, *R*, and *V* represent the fractions of the population that are susceptible, infected, recovered, and vaccinated, respectively, such that S+V+I+R=1. The transmission rate, β(t), is a *T*-periodic non-negative function representing seasonality, where *T* is typically one year. The parameters γ, $\delta _R$, and $\delta _V$ are the rates of recovery, waning of natural immunity, and waning of vaccine-induced immunity, respectively, while μ is the demographic turnover rate (births and deaths). All the above rate parameters have a dimension of $\textrm{time}^{-1}$. Fig. 1 presents a flowchart of the model.

The control variable is the *T*-periodic instantaneous vaccination rate, η(t), with a dimension of $\textrm{time}^{-1}$.

We assume here that vaccination is administered to all non-infectious individuals (*S*, *R*, and *V* compartments). By this assumption, the mean number of vaccine doses administered over a period *T*, divided by the population size, is given by

2 $$\begin{aligned} \bar{\eta }:=\frac{1}{T}\int _0^T \eta (t)[S(t)+R(t)+V(t)]dt. \end{aligned}$$

so that $\bar{\eta }T$ is the total number of vaccines administered per period, divided by the population size.

The rationale for our assumption that individuals to be vaccinated are selected at random among the non-infected individuals is that we are concerned with the case in which immunity (following vaccination or recovery) is temporary - necessitating repeated vaccination to maintain immunity. However, the duration of time for which a vaccinated (or recovered) individual remains protected, that is remains in class *V* (or *R*), is a random variable which is exponentially distributed with mean $\delta _V^{-1}$ ($\delta _R^{-1})$. Therefore it is not possible to determine whether a randomly chosen individual is in class *S*, except by carrying out pre-screening for sero-status, which is only practical and cost-effective when the cost of vaccination significantly exceeds that of pre-screening (Alp et al. 2012; Jacobs et al. 2003). Thus assuming that vaccination is restricted to individuals in class *S* is not realistic in the context of repeated vaccination. Table 1 summarizes the state variables and parameters of the model.

**Table 1:** State variables, control variables, and parameters of the SIRVS model

Symbol	Biological Meaning	Unit/Dim.	Baseline
**State Variables**			
*S*	Fraction of susceptible individuals	Dimensionless	−
*I*	Fraction of infected individuals	Dimensionless	−
*R*	Fraction of recovered individuals	Dimensionless	−
*V*	Fraction of vaccinated individuals	Dimensionless	−
**Control Variable**			
η(t)	Instantaneous vaccination rate	$\textrm{year}^{-1}$	Periodic
**Model Parameters**			
*T*	Period duration (seasonality)	year	1 (One year)
β(t)	Transmission rate (seasonality)	$\textrm{year}^{-1}$	Periodic (Seasonality)
γ	Rate of recovery	$\textrm{year}^{-1}$	52 (Mean infectious duration - 1 week)
$\delta _R$	Waning rate of natural immunity	$\textrm{year}^{-1}$	1 (Mean immunity duration - 1 year)
$\delta _V$	Waning rate of vaccine-induced immunity	$\textrm{year}^{-1}$	1 (Mean immunity duration - 1 year)
μ	Demographic turnover rate (birth/death)	$\textrm{year}^{-1}$	1/80 (Average lifespan - 80 years)

In Onyango and Müller (2014), Onyango and Müller treat the problem of optimal periodic vaccination in a seasonal environment, but their model assumes that vaccination and infection induce life-long immunity (that is, $\delta _V=\delta _R=0$). Within this framework, it is natural to assume, as is done in Onyango and Müller (2014), that only susceptible individuals are vaccinated, since the susceptible individuals are easily recognized as those who have not been previously vaccinated or infected. Under this assumption, the expression (2) for the mean number of vaccination doses administered reduces to $\bar{\eta }=\frac{1}{T}\int _0^T \eta (t)S(t)dt$. Interestingly, the nature of the optimal vaccination profiles obtained under the assumption of Onyango and Müller (2014) is quite different from that of the profiles obtained under our alternative assumption; a comparison is provided in Sect. 9.

It should be recognized, however, that our assumption that all non-infected individuals are eligible for vaccination is an idealization, a higher fidelity model should account for the fact that recently-vaccinated individuals are less likely to be re-vaccinated. Our formulation is thus dictated in part by mathematical tractability, see section 9 for discussion of this and other desired directions for extending our investigation.

### Optimization Criterion

Our aim is to choose the vaccination profile η(t) optimally to achieve long-term local elimination of the disease with a minimal vaccination effort per year. Let us first focus on the conditions required to achieve such elimination. In our deterministic framework, we model this by requiring the disease-free state to be globally stable. This ensures that if the disease is reintroduced into the population, the disease will die out with time.

The disease-free state is obtained by substituting I≡0 and R≡0 in (1) yielding

3 $$\begin{aligned} \begin{aligned}S^\prime (t)&=\mu +\delta _V V -(\eta (t)+\mu )S,\\ V^\prime (t)&=\eta (t)S-(\delta _V+\mu ) V. \end{aligned} \end{aligned}$$

Since S(t)+V(t)=1 when I=R=0, and setting

$$\begin{aligned} \delta =\delta _V+\mu , \end{aligned}$$

the system (3) reduces to the single linear equation

4 $$\begin{aligned} S'=\delta (1-S) -\eta (t)S,\end{aligned}$$

and the time-averaged vaccination rate (2) reduces to

$$ \bar{\eta }=\frac{1}{T}\int _0^T \eta (t)dt. $$

Equation (4) has a unique *T*-periodic solution $S_p(t)$, and all its solutions tend asymptotically to this periodic solution. Explicitly, this periodic solution is given by

5 $$\begin{aligned} \begin{aligned} S_p(t)=\frac{\delta }{1-e^{-\int _0^T[\eta (s)+\delta ]ds}}\left[ \int _0^t e^{-\int _r^t [\eta (s)+\delta ]ds}dr+e^{-\int _0^T [\eta (s)+\delta ]ds}\int _t^T e^{-\int _r^t[\eta (s)+\delta ]ds}dr\right] . \end{aligned} \end{aligned}$$

The disease-free equilibrium is locally (and globally) stable - that is the disease is eliminated over time - if and only if the basic reproduction number, $\mathcal {R}[\eta ]$ satisfies (Rebelo et al. 2012; Doutor et al. 2016)

6 $$\begin{aligned} \mathcal {R}[\eta ]\doteq \frac{1}{(\gamma +\mu )T}\int _0^T \beta (t)S_p(t)dt<1. \end{aligned}$$

Doutor et al. (2016) also addressed the optimal elimination of a seasonal disease using a periodic vaccination rate. However, their approach imposes a more stringent condition on the vaccination profile: specifically, that the *instantaneous* reproduction number,

$$\begin{aligned} \mathcal {R}(t) = \frac{\beta (t)S(t)}{\gamma + \mu }, \end{aligned}$$

must remain below unity for all *t*. This condition ensures that the prevalence of infection decreases monotonically. In contrast, as in Onyango and Müller (2014), we require only that the threshold parameter $\mathcal {R}[\eta ]$, which for the present model corresponds to the *time average* of the instantaneous reproduction number, is less than one. While the condition $\mathcal {R}[\eta ] < 1$ guarantees long-term disease eradication, the number of infected individuals may not exhibit monotonic decay during the transient phase. This distinction in stability criteria necessitates fundamentally different analytical methods and leads to divergent results. Notably, the stricter criterion in Doutor et al. (2016) necessarily entails a larger annual vaccine investment than the threshold condition $\mathcal {R}[\eta ] < 1$.

It is instructive to examine condition (6) for a constant vaccination rate $\eta (t)\equiv \eta ^u$ so that the mean vaccination rate is $\bar{\eta }=\eta ^u$. In this case, we have $S_p(t)\equiv \frac{\delta }{\delta +\bar{\eta }}$ so that condition (6) reads

7 $$\begin{aligned} \bar{\eta }>\bar{\eta }_c:=\left( \frac{\bar{\beta }}{\gamma +\mu }-1\right) \delta =\left( {\mathcal {R}}_0-1\right) \delta ,\quad \bar{\beta }=\frac{1}{T}\int _0^T\beta (s)\,ds, \end{aligned}$$

where ${\mathcal {R}}_0=\frac{\beta }{\gamma +\mu }$ is the reproductive number in the absence of vaccination. That is, with a constant vaccination profile, condition (6) is satisfied if the (mean) vaccination rate $\bar{\eta }$ exceeds $\bar{\eta }_c$.

We ask whether, by choosing an appropriate time-periodic vaccination profile η(t), elimination can be achieved using a lower mean vaccination rate. In other words, can one find η(t) for which $\bar{\eta } < \bar{\eta }_c,$ and for which $\mathcal {R}[\eta ]<1$? If so, what is the *minimal* mean vaccination rate $\bar{\eta }$ that can achieve elimination? To address this question, it is useful to define a dual problem, in which we fix the time-averaged vaccination rate $\bar{\eta }$ and seek to minimize the reproductive number:

For a given average vaccination rate $\bar{\eta }$, find the *T*-periodic profile, satisfying $\frac{1}{T}\int _0^T \eta (t)dt=\bar{\eta }$, which minimizes the reproductive number $\mathcal {R}[\eta ]$. This optimal profile will be denoted $\eta ^\textrm{opt}(t;\bar{\eta })$.

By solving this problem, we can also solve the problem of achieving elimination using a minimal amount of vaccine: we define the function

8 $$\begin{aligned} \mathcal {R}^\textrm{min}(\bar{\eta })= \mathcal {R}[\eta ^\textrm{opt}(t;\bar{\eta })], \end{aligned}$$

which gives the lowest possible reproductive number for a given average effort $\bar{\eta }$. This function is obviously monotone decreasing. The minimal effort required for elimination, $\bar{\eta }^\textrm{min}$, can then be found by solving the equation

$$\mathcal {R}^\textrm{min}(\bar{\eta }^\textrm{min})= 1. $$

We will thus concentrate on the problem of minimizing $\mathcal {R}[\eta ]$, employing a given annual amount of vaccines.

### Optimization Over a Space of Measures

To formalize the problem, we must specify the set of allowed vaccination profiles η(t) over which optimization is to be performed. Here, it is important for us that the set of allowed profiles includes, for example, the case of pulse vaccination, which corresponds to a periodic series of Dirac δ-functions:

$$\begin{aligned} \eta (t)=\bar{\eta }\sum _{k\in \mathbb {Z}} \delta _0(t-t_0+kT). \end{aligned}$$

To do this, we follow Gavish and Katriel (2026) and allow our vaccination profiles to be *measures*, rather than functions. Thus, we will consider vaccination profiles defined by non-negative Borel measures on the real line $$\mathbb {R}$$ which are locally finite (meaning that the measure of bounded sets is finite), where the quantity $$m([t_1,t_2))$$ describes the vaccination effort made during the time interval $$[t_1,t_2)$$ (the standard definitions and results from measure theory employed below can be found in, *e.g.*, Rudin (1987), Ch. 1-2,6-7). Since we are interested in periodic vaccination, we restrict ourselves to measures which are *T*-periodic, in the sense that

$$ m([t_1+T,t_2+T))=m([t_1,t_2)),\qquad \forall t_1<t_2. $$

The space of all locally finite non-negative *T*-periodic Borel measures will be denoted by ${\mathcal {M}}$. The space ${\mathcal {M}}$ is our generalized space of vaccination profiles. We note that, because of the *T*-periodicity, a measure $m\in {\mathcal {M}}$ is determined by its restriction to the interval [0, *T*).

With any measure $m\in {\mathcal {M}}$ we can associate a cumulative function

9 $$\begin{aligned} \alpha _{m}(t)\doteq m([0,t)),\qquad t\in [0,T],\end{aligned}$$

and the mapping $m\rightarrow \alpha _{m}$ defines a one-to-one correspondence between such measures and monotone non-decreasing left-continuous functions on [0, *T*] satisfying $\alpha _m(0)=0$. In our case $\alpha _{m}(t)$ is the number of vaccinations administered in the time interval [0, *t*), divided by the population size. We recall also that the measure m is said to be *absolutely continuous* if the measure m(S)=0 whenever *S* is a set of Lebesgue measure 0. The Lebesgue equivalent of the fundamental theorem of calculus says that a measure $m\in {\mathcal {M}}$ is absolutely continuous if and only if it can be represented in the form

10 $$\begin{aligned} m([t_1,t_2))=\int _{t_1}^{t_2} \eta (t)dt,\qquad \forall t_1<t_2,\end{aligned}$$

where the density η is a locally $L^1$ (that is $L^1$ on bounded intervals) function, equal to the derivative $\alpha _{m}^\prime$ (almost everwhere with respect to the Lebesgue measure), and *T*-periodicity of the measure *m* is equivalent to *T*-periodicity of the density η. Thus the vaccination profiles η(t) considered previously, where η(t) is a non-negative locally $L^1$ *T*-periodic function, are embedded as a subset of ${\mathcal {M}}$, by identifying η with the absolutely continuous measure m defined by (10), in which case the corresponding cumulative vaccination function is given by

11 $$\begin{aligned} \alpha _{m}(t)=\int _0^t \eta (t)dt,\qquad t\in [0,T].\end{aligned}$$

However, the space ${\mathcal {M}}$ also contains measures which are not absolutely continuous: for example, if the cumulative function $\alpha _m(t)$ is discontinuous at a point $t^*\in [0,T)$, then the measure *m* has atomic components at points $t^*+kT$ ($k\in \mathbb {Z}$), corresponding to pulses of vaccination of size $\alpha _m(t^*+)-\alpha _m(t^*-)$ given at these times.

We now generalize the definition of the functional $\mathcal {R}$ to an arbitrary measure $m\in {\mathcal {M}}$, in the unique way that is consistent with the definition (6).

In view of (11), the expression (5) can be generalized to

12 $$\begin{aligned} \begin{aligned}&S_p(t)=\frac{\delta }{1-e^{-[\alpha _{m}(T)+\delta T]}} \\ &\quad \left[ \int _0^t e^{-[\alpha _{m}(t)-\alpha _{m}(r)+\delta (t-r)]}dr+e^{-[\alpha _{m}(T)+\delta T]}\int _t^T e^{-[\alpha _{m}(t)-\alpha _{m}(r)+\delta (t-r)]}dr\right] . \end{aligned} \end{aligned}$$

Substituting this expression into (6) yields the value of $\mathcal {R}$, which can be explicitly written as

13 $$\begin{aligned} \begin{aligned} \mathcal {R}[m]&=\frac{1}{(\gamma +\mu ) T}\cdot \frac{\delta }{1-e^{-[\alpha _{m}(T)+\delta T]}}\times \\ &\int _0^T\beta (t)\left[ \int _0^t e^{-[\alpha _{m}(t)-\alpha _{m}(r)+\delta (t-r)]}dr+e^{-[\alpha _{m}(T)+\delta T]}\int _t^T e^{-[\alpha _{m}(t)-\alpha _{m}(r)+\delta (t-r)]}dr\right] dt. \end{aligned} \end{aligned}$$

(13) coincides with (6) when *m* is an absolutely continuous measure with density η. Moreover, the functional $\mathcal {R}$ defined by (13) is continuous on the space ${\mathcal {M}}$ with respect to the topology of weak convergence of measures (see (Gavish and Katriel (2026), Lemma 2)). Since the set of absolutely continuous measures is dense with respect to the weak topology, it follows that (13) is the *unique* way to extend (6) to all measures.

The constraint $\frac{1}{T}\int _0^T \eta (t)dt=\bar{\eta }$ translates to

14 $$\begin{aligned} \frac{1}{T}m([0,T))=\bar{\eta }.\end{aligned}$$

Our optimization problem is thus formulated as,

#### Problem 1

Given $\bar{\eta }>0$, find, among all vaccination profiles $m\in {\mathcal {M}}$ which satisfy (14), the one for which $\mathcal {R}[m]$ is minimal.

### Existence and Uniqueness of the Optimal Vaccination Profile

The following result guarantees that Problem 1 has a unique solution.

#### Theorem 2

Assume β(t) is a non-negative *T*-periodic function, which is locally $L^1$. Then, Problem 1 has a unique solution.

This theorem is proved, in a somewhat more general form, in (Gavish and Katriel 2026, Theorem 1, Section 2). The following Lemma shows that a better outcome cannot be achieved by considering an *nT*-periodic profile for n≥2, so that it is justified to restrict our study to periodic profiles with the same period as that of the seasonality.

#### Lemma 3

Assume β(t) is locally $L^1$, non-negative and *T*-periodic, and let $m_n$ be a non-negative Borel measure on the real line which is nT-periodic

$$ m_n([t_1+nT,t_2+nT))=m_n([t_1,t_2))\qquad \forall t_1<t_2, $$

and which solves Problem 1 with *T* replaced by *nT*. Then $m_n$ is, in fact, *T*-periodic.

#### Proof

Since β(t) is *T*-periodic, the measure defined by

$$\begin{aligned} \tilde{m}_n([t_1,t_2))=m_n([t_1+T,t_2+T)) \end{aligned}$$

is also a solution of Problem 1 with *T* replaced by *nT*. Therefore by the uniqueness of the solution of Problem 1 (Theorem 2), we must have $\tilde{m}_n=m_n$, which means

$$\begin{aligned} m_n([t_1+T,t_2+T))=m_n([t_1,t_2)), \end{aligned}$$

so that $m_n$ is in fact *T*-periodic. □

We will denote the unique solution to Problem 1 by $m^\textrm{opt}$, and call it the optimal vaccination profile. We will also denote by $\Omega (m^\textrm{opt})$ the support of this measure, that is, the set of values *t* for which $m^\textrm{opt}(t-\epsilon ,t+\epsilon ))>0$ for all ϵ>0. The set $\Omega (m^\textrm{opt})$ is the set of times at which vaccines are administered. By definition, it follows that it is a closed set and, by the periodicity of $m^\textrm{opt}$ it is a *T*-periodic set in the sense that $t\in \Omega (m^\textrm{opt})$ implies $t+kT\in \Omega (m^\textrm{opt})$ for all $k\in \mathbb {Z}$. When $m^\textrm{opt}$ is absolutely continuous with density $\eta ^\textrm{opt}$, we will also use the notation $\Omega (\eta ^\textrm{opt})$ for the support; in this case $\Omega (\eta ^\textrm{opt})$ is simply the set in which $\eta ^\textrm{opt}$ does not vanish. We will also denote by $\alpha ^\textrm{opt}$ the cumulative effort function corresponding to $m^\textrm{opt}$, see (9), and by $S^\textrm{opt}(t)$ the periodic function describing the fraction of susceptibles under the vaccination profile $m^\textrm{opt}$ in the disease-free state, which is computed using (12).

In what follows, we aim to understand the structure of the solution $m^\textrm{opt}$ and its dependence on the periodic transmission function β(t) and on the parameters $\bar{\eta },\delta$ of the problem.

## Characterization of the Optimal Vaccination Profile

We now turn to our central result, which explicitly characterizes the solution of problem 1. Although Problem 1 is defined over the entire space of measures ${\mathcal {M}}$, we find that under certain regularity conditions on the transmission profile β(t), the optimal solution $m^\textrm{opt}$ is, in fact, an absolutely continuous measure. Moreover, as stated in the following theorem, an explicit expression for the density of this measure can be provided.

### Theorem 4

Assume β(t)>0 is a *T*-periodic function that is absolutely continuous on every bounded closed interval and differentiable at all points apart from a countable set. Then, the solution $m^\textrm{opt}$ of Problem 1 is an absolutely continuous measure, with density $\eta ^\textrm{opt}$, that is, for all $t_1<t_2$,

$$\begin{aligned} m^\textrm{opt}([t_1,t_2))=\int _{t_1}^{t_2} \eta ^\textrm{opt}(t)dt, \end{aligned}$$

and the density is given by

15 $$\begin{aligned} \eta ^\textrm{opt}(t)={\left\{ \begin{array}{ll} \eta ^*(t) & t\in \Omega (\eta ^\textrm{opt}),\\ 0 & t\not \in \Omega (\eta ^\textrm{opt}), \end{array}\right. } \end{aligned}$$

where

16 $$\begin{aligned} \eta ^*(t)=\frac{\sqrt{\beta (t)}}{\lambda }+\frac{1}{2}\frac{\beta ^\prime (t)}{\beta (t)}-\delta , \end{aligned}$$

with λ given by

17 $$\begin{aligned} \lambda =\frac{ \int _{\Omega }\sqrt{\beta (s)}ds}{T\bar{\eta }+\delta |\Omega |-\frac{1}{2}\int _{\Omega }\frac{\beta '(s)}{\beta (s)}ds}, \end{aligned}$$

where $\Omega =\Omega (\eta ^\textrm{opt})\cap [0,T)$, and |Ω| is the Lebesgue measure of Ω.

The proof of Theorem 4 is given in Gavish and Katriel (2026): the result is obtained by applying (Gavish and Katriel 2026, Theorem 2) with c(t)=δ and w(t)=β(t).

Theorem 4 says that the optimal vaccination profile is composed of time intervals in which some vaccination effort is made ($\eta ^\textrm{opt}(t)>0$) - the union of these intervals is the set $\Omega (\eta ^\textrm{opt})$, and time intervals in which no vaccinations are administered ($\eta ^\textrm{opt}(t)=0$). The theorem provides an explicit expression for the optimal vaccination profile on its support, though it does not provide an analytical description of the support set $\Omega (\eta ^\textrm{opt})$. However, when $\bar{\eta }$ is sufficiently large, then, as will be shown in subsection 4.2, we have $\Omega (\eta ^\textrm{opt})=\mathbb {R}$, in which case our result provides a fully explicit expression for $\eta ^\textrm{opt}$. To obtain a full description of the optimal profile when $\Omega (\eta ^\textrm{opt})\ne \mathbb {R}$, we subsequently consider numerically-computed examples.

### Numerical Examples

We provide numerical examples to illustrate the contents of Theorem 4. We use a numerical computation based on the direct minimization of a discretization of the nonlinear functional $\mathcal {R}$ to obtain the cumulative vaccination effort: Note that in (13), the functional $\mathcal {R}$ is formulated in terms of the cumulative effort function, $\alpha _m(t)$, which is, by definition, a nondecreasing monotonic function, and this constraint is imposed in the numerical optimization scheme. The vaccination profile $\eta ^\textrm{opt}$ is then obtained as the discrete derivative of $\alpha ^\textrm{opt}$. Thus, an interval with no vaccination, $\eta ^\textrm{opt}(t)=0$, corresponds to a flat segment of $\alpha ^\textrm{opt}(t)$, a continuous vaccination rate, that is an interval on which $m^\textrm{opt}$ is absolutely continuous with $\eta ^\textrm{opt}(t)>0$ corresponds to a segment in which in $\alpha ^\textrm{opt}(t)$ is continuous and strictly increasing, and a vaccination pulse, i.e., a Dirac delta component of $m^\textrm{opt}$, corresponds to a jump discontinuity in $\alpha ^\textrm{opt}(t)$. Details of the numerical method are given in (Gavish and Katriel 2026, Appendix B). The codes used to compute the data and produce the graphs in this manuscript are available; see supplementary material.

To model seasonal diseases such as influenza, where the transmission rate changes throughout the year, we assign T=1 to denote one year. Initially, we set δ=1, indicating that the mean duration of vaccine-induced immunity is one year, and later explore other δ values ranging from δ=0.1 (mean duration of immunity is 10 years) to δ=4 (mean duration of immunity is three months). The default scenario assumes a mean vaccination rate of $\bar{\eta }=0.3$ per period, which means that the number of vaccinations administered annually is 30% of the population size. We will also analyze scenarios with values of $\bar{\eta }$ from $\bar{\eta }=0.1$ (10% vaccination annually) to $\bar{\eta }=2$ (annual amount of vaccinations is twice the entire population size, so that, on average, each individual is vaccinated twice a year). Parameters such as the recovery rate γ, and the mean transmission rate $\bar{\beta }$ do not influence the solution to Problem 1 since they only rescale the cost functional $\mathcal {R}$, as defined by (6). However, for studying the solutions of the SIRVS system (1), we specify μ=1/80 to reflect an average lifespan of 80 years, set $\left. \gamma =52\right.$ to indicate a mean duration of one week from infection to recovery, and define $\bar{\beta }=\frac{1}{T}\int _0^T\beta (s)ds=(\gamma +\mu ) {\mathcal {R}}_0$ where $\left. {\mathcal {R}}_0=2.1\right.$.

**Fig. 2:**
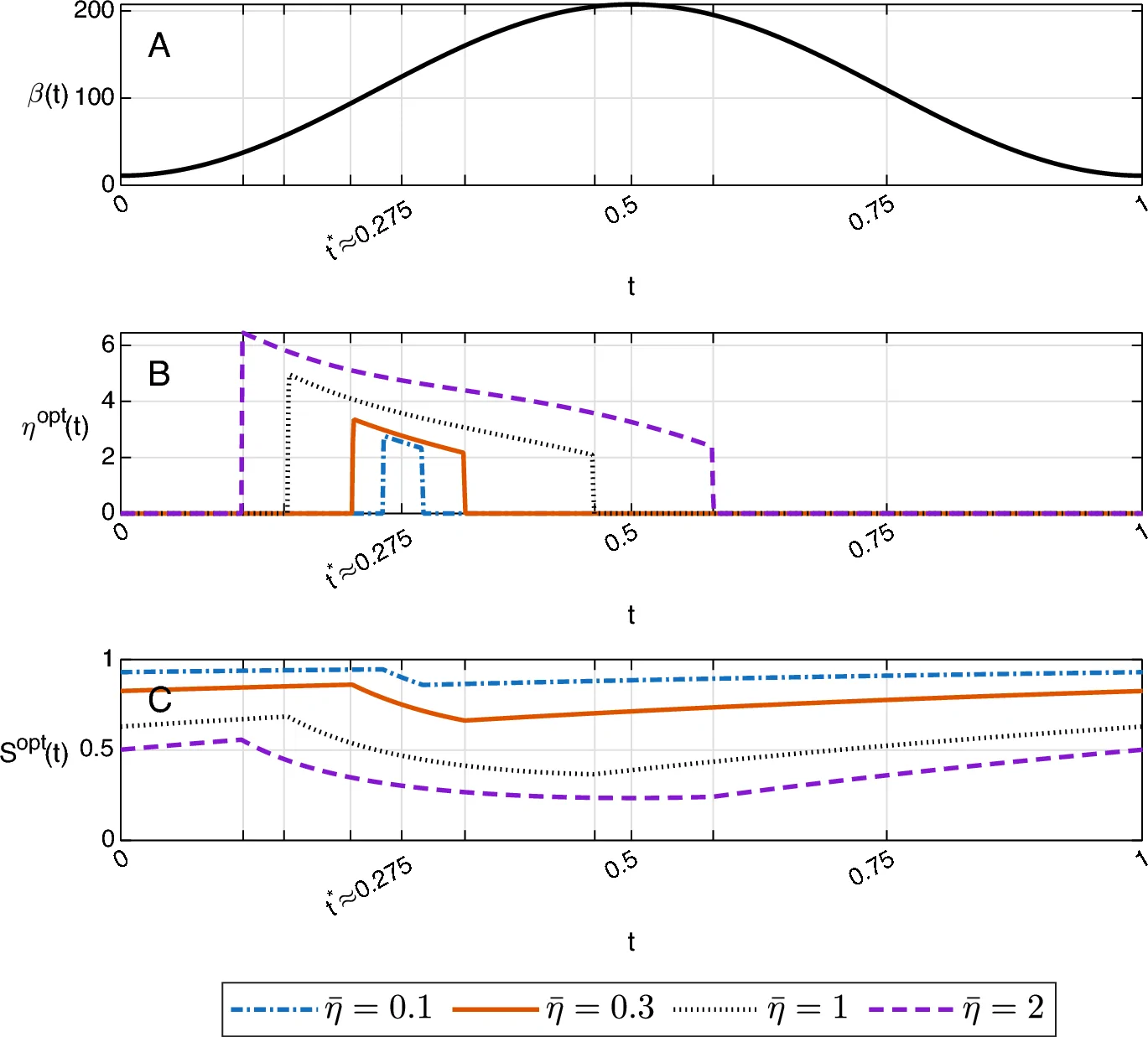
Optimal vaccination profile $\eta ^\textrm{opt}(t)$ for $\beta (t)=\bar{\beta }\cdot (1-0.9\cos (2\pi t))$ with $\left. \bar{\beta }=2.1\cdot (\gamma +\mu )\right.$, and δ=1 and for different values of $\bar{\eta }$: $\bar{\eta }=0.1$ (blue dash-dotted curve), $\bar{\eta }=0.3$ (red solid curve), $\bar{\eta }=1$ (black dotted curve) and $\bar{\eta }=2$ (purple dashed curve). **A** β(t). **B** $\eta ^\textrm{opt}$ and **C** $S^\textrm{opt}$

In Fig. 2, we present an example with the 1-periodic transmission profile

18 $$\begin{aligned} \beta (t)=\bar{\beta }\cdot (1-a\cos (2\pi t)),\quad \bar{\beta }=(\gamma +\mu ) {\mathcal {R}}_0, \end{aligned}$$

with a=0.9, which attains its peak at t=0.5, as shown in Fig. 2A. We begin with the scenario where δ=1 and $\bar{\eta }=0.3$. The optimal vaccination profile involves administering vaccines during the interval $0.22\lesssim t\lesssim 0.33$ (roughly one month) and refraining from vaccination outside this window. Equation (16) of Theorem 4 characterizes the vaccination profile within this interval as given by the expression

19 $$\begin{aligned} \eta ^*(t)=\frac{\sqrt{1-a\cos (2\pi t)}}{\lambda }-\frac{a\pi \sin (2\pi t)}{1-a\cos (2\pi t)}-\delta ,\end{aligned}$$

but does not provide any information on the duration and location of the vaccination interval $\Omega (\eta ^\textrm{opt})$. Since knowledge of this interval is required to obtain the value λ using (17), we also do not have an analytical expression for λ. However, after solving the problem numerically and finding $\Omega (\eta ^\textrm{opt})$, we verify that the solutions agrees with the expression (19), with the value of λ which is determined by (17).

As the values of $\bar{\eta }$ decrease, the vaccination interval contracts. For instance, with $\bar{\eta }=0.1$, the interval is approximately $0.25 \lesssim t \lesssim 0.29$ (roughly two weeks) as depicted by the blue dash-dotted curve in Fig. 2B. Conversely, the intervals broaden to $0.16 \lesssim t \lesssim 0.46$ (about 4 months) and $0.11 \lesssim t \lesssim 0.58$ (nearly half a year) when $\bar{\eta }=1$ and $\bar{\eta }=2$, respectively, represented by the black dotted and dashed purple curves in the same figure. Subsequently, we will explore how $\eta ^\textrm{opt}$ varies with $\bar{\eta }$.

## Dependence of the Optimal Vaccination Profile on the Average Vaccination Rate $\bar{\eta }$

In the following analysis, we examine two extreme scenarios: one with a low average vaccination rate ($\bar{\eta }\rightarrow 0$) and another with a high average vaccination rate (large $\bar{\eta }$). We work under the assumptions outlined in Theorem 4, ensuring that $m^\textrm{opt}$ is absolutely continuous, characterized by the density $\eta ^\textrm{opt}$. For clarity, it is beneficial to explicitly incorporate the parameter $\bar{\eta }$ into the notation for the optimal profile, denoting the optimal profile associated with $\bar{\eta }$ by $\eta ^\textrm{opt}(t;\bar{\eta })$.

### Small $\bar{\eta }$

**Fig. 3:**
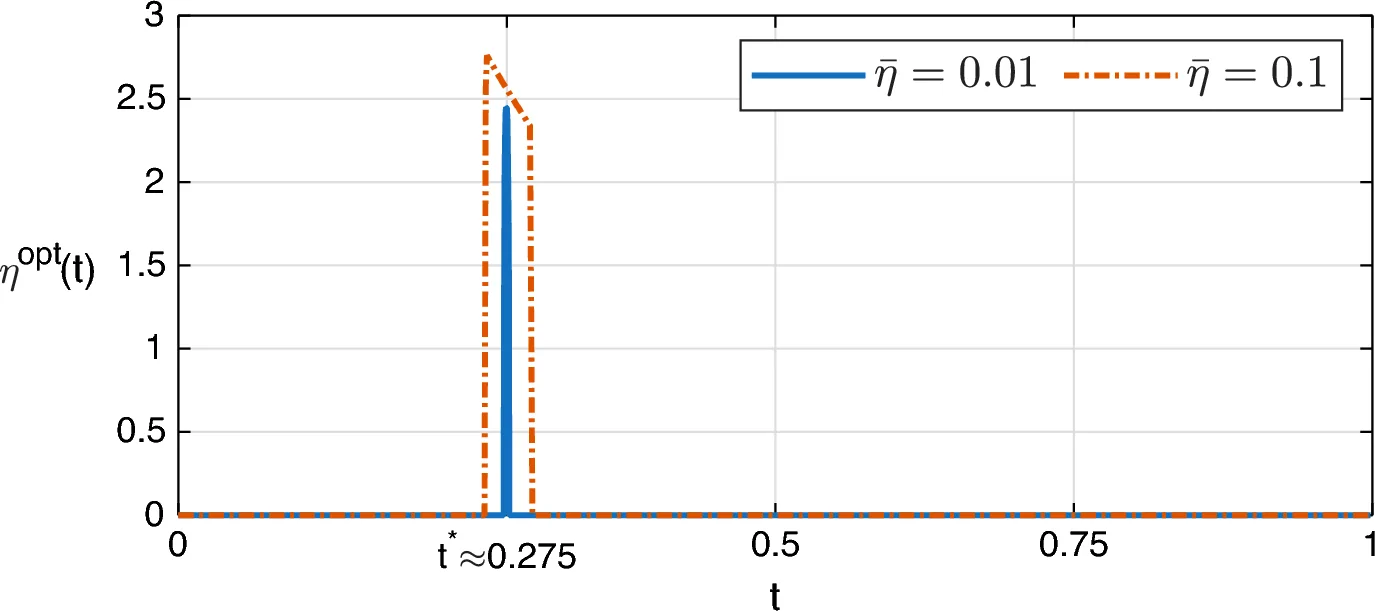
Optimal vaccination profile $\eta ^\textrm{opt}$ for the same seasonality as in Fig. 2, for smaller values of $\bar{\eta }$: $\bar{\eta }=0.1$ (dash-dotted red curve) and $\bar{\eta }=0.01$ (solid blue curve). Note that the graph of $\eta ^\textrm{opt}(t;\bar{\eta }=0.1)$ is the same graph as in Fig. 2C, but presented in with different y-axis scaling

As illustrated in Fig. 2, the vaccination interval narrows as $\bar{\eta }$ decreases. In the limit of a scarce vaccine supply, $\bar{\eta }\rightarrow 0$, the vaccination interval narrows and concentrates around a single time point, $t^*$. The optimal strategy thus converges to pulse vaccination where the entire yearly vaccine stockpile is administered on a particular date $t^*$ within the year. This phenomenon is further demonstrated in Fig. 3, which shows the optimal vaccination profile for the reduced value $\bar{\eta }=0.01$.

The following result, which follows from (Gavish and Katriel 2026, Theorem 4), shows that this observation is valid in general, and also provides a way of identifying the value $t^*$.

#### Theorem 5

Under the assumptions of Theorem 4, define the function

20 $$\begin{aligned} f(t)=[e^{\delta t}-1]\int _t^T \beta (r)e^{-\delta r } dr-[1-e^{\delta (t-T)}] \int _0^t \beta (r)e^{-\delta r }dr, \end{aligned}$$

which is *T*-periodic. Let

$$\begin{aligned} f_{\max }=\max _{t\in \mathbb {R}} f(t), \end{aligned}$$

and, for ϵ>0, define the set

$$\begin{aligned} M_{\epsilon }=\{ t\in \mathbb {R}\;|\; f(t)>f_{\max }-\epsilon \}. \end{aligned}$$

Then, for any ϵ>0, there exists a value $\bar{\eta }(\epsilon )$ such that

$$\begin{aligned} \bar{\eta }\in (0,\bar{\eta }(\epsilon ))\quad \Rightarrow \quad \Omega (\eta ^\textrm{opt}(\cdot ;\bar{\eta }))\subset M_\epsilon . \end{aligned}$$

Thus, whenever the global maximum point $t^*$ of the function *f*(*t*) defined by (20) is unique in [0, *T*), which will happen generically, the set $\Omega (\eta (\cdot ;\bar{\eta }))$ concentrates near the point $t^*$.

For the 1-periodic transmission profile (18) used in Fig. 2, the function *f*(*t*) can be explicitly calculated and maximized, to yield the expression

21 $$\begin{aligned} t^*=\frac{1}{2}\left[ 1 - \frac{1}{\pi }\arctan \left( \frac{2\pi }{\delta }\right) \right] . \end{aligned}$$

For the parameter value δ=1 used in Fig. 3, we find $t^*\approx 0.275$, which agrees with the value to which the vaccination interval converges in the numerical example shown in Fig. 2. It is important to note that in this scenario, a large value of δ, indicating rapid decay of immunity after vaccination, brings $t^*$ close to $\frac{1}{2}$, suggesting optimal vaccination around the peak of transmissibility. In contrast, when δ is small, meaning immunity to vaccination is prolonged, $t^*$ is close to $\frac{1}{4}$, making it optimal to vaccinate about a quartile before peak transmissibility.

### Large Values of $\bar{\eta }$

The following theorem shows that, given a sufficient amount of vaccines, it is optimal to administer vaccines, without pause, throughout the year, that is, $\Omega (\eta ^\textrm{opt})=\mathbb {R}$. In this case, the optimal vaccination profile is explicitly characterized as follows

#### Theorem 6

Under the assumptions of Theorem 4, let

$$ \bar{\eta }_m=\sup _{t\in [0,T]}\left[ \frac{\delta }{\sqrt{\beta (t)}}-\frac{1}{2}\frac{\beta ^\prime (t)}{\beta ^{3/2}(t)}\right] \int _0^T\sqrt{\beta (s)}ds-\delta . $$

Then, for any $\bar{\eta }>\bar{\eta }_m$, the solution $m^\textrm{opt}$ of Problem 1 has density $\eta ^\textrm{opt}$ which satisfies $\eta ^\textrm{opt}(t)>0$ for all *t*, and is explicitly given by

22 $$\begin{aligned} \eta ^\textrm{opt}(t)=\frac{(\delta +\bar{\eta })\sqrt{\beta (t)}}{\frac{1}{T}\int _0^T\sqrt{\beta (s)}ds}+\frac{1}{2}\frac{\beta ^\prime (t)}{\beta (t)}-\delta , \end{aligned}$$

and the corresponding solution of (5) is

23 $$\begin{aligned} S_{opt}(t)= \frac{\frac{1}{T}\int _0^T \sqrt{\beta (s)}ds}{\delta +\bar{\eta }}\cdot \frac{1}{\sqrt{\beta (t)}}. \end{aligned}$$

The optimal value of $\mathcal {R}$ is given by

24 $$\begin{aligned} \mathcal {R}[m^\textrm{opt}]=\frac{1}{\gamma +\mu }\cdot \frac{1}{\bar{\eta }+\delta }\left( \frac{1}{T}\int _0^T \sqrt{\beta (s)} ds\right) ^2. \end{aligned}$$

#### Proof

Note that, as is easily checked, the condition $\bar{\eta }>\bar{\eta }_m$ is equivalent to the condition that the function defined by (22) is everywhere positive, so that the general expression (15) given in Theorem 4 immediately implies that if $\Omega (\eta ^\textrm{opt})=\mathbb {R}$, that is if $\eta ^\textrm{opt}$ is everywhere positive, then $\bar{\eta }>\bar{\eta }_m$. To complete the proof of the theorem, it is necessary to show the converse - that if the expression on the right-hand side of (22) is positive, then it is equal to $\eta ^\textrm{opt}$. This follows from (Gavish and Katriel 2026, Lemma 18). The expression (23) is computed by substituting (22) in in (5). The expression (24) is obtained by substituting (23) in the expression (6) for $\mathcal {R}$. □

**Fig. 4:**
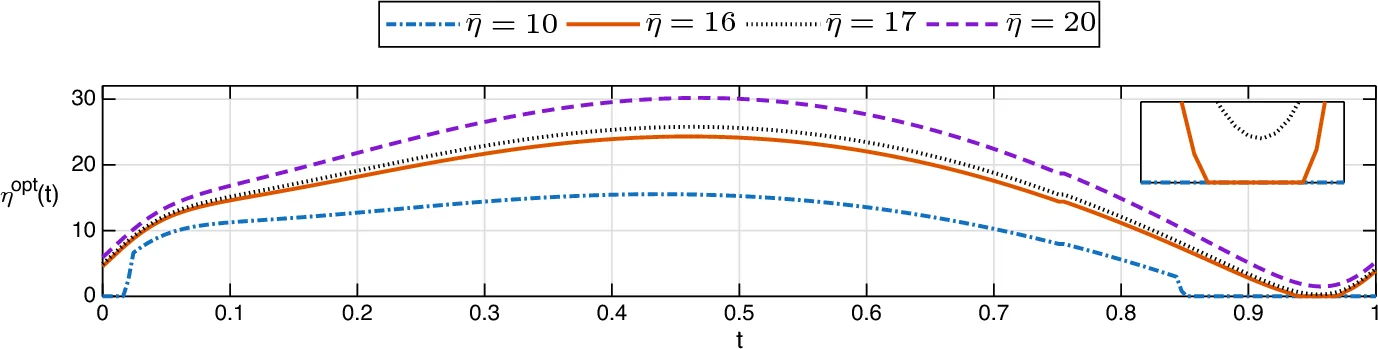
Optimal vaccination profile $\eta ^\textrm{opt}$ for the same seasonality as in Fig. 2, for larger values of $\bar{\eta }$: $\bar{\eta }=10$ (dash-dotted blue curve), $\bar{\eta }=16$ (solid red curve), $\bar{\eta }=17$ (dotted black curve) and $\bar{\eta }=20$ (dashed purple curve)

Figure 4 illustrates the result of Theorem 6 by presenting the optimal vaccination profile $\eta ^\textrm{opt}$ for the same parameters as in Fig. 2 but for large values of $\bar{\eta }$. In this case, $\bar{\eta }_m\approx 16.36$. As expected, for $\bar{\eta }=10$, we observe that the optimal vaccination profile includes a substantial period without vaccination, see the dash-dotted blue curve. For $\bar{\eta }=16$, shy of $\bar{\eta }_m\approx 16.36$, we observe that the optimal vaccination profile includes a short period without vaccination (0.94<t<0.97), see the solid red curve. For values of $\bar{\eta }>\bar{\eta }_m$, the optimal vaccination profile is positive for all t∈[0,1], see dotted black curve for $\bar{\eta }=17$ and dashed purple curve for $\bar{\eta }=20$.

In the previous example, we found that $\bar{\eta }_m\approx 16.36$, indicating that the number of vaccines administered per year is more than 16 times the population size. Clearly, this scenario is unrealistic. However, as the strength of the seasonality decreases, the value of $\eta _m$ approaches more practical levels. For the profile (18), we compute that $\eta _m\approx 0.83$ when a=0.25, and $\left. \eta _m\approx 2.32\right.$ when a=0.5.

Note that when $\beta (t)=\bar{\beta }$ is constant, we have $\eta _m=0$, which implies that expression (22) for $\eta ^\textrm{opt}$ is valid for any $\bar{\eta }>0$, and this expression reduces to $\eta ^\textrm{opt}(t)=\bar{\eta }$, so that we have

#### Corollary 7

If $\beta (t)=\bar{\beta }$ then, for any $\bar{\eta }>0$, the solution to Problem 1 is $\eta ^\textrm{opt}(t)=\bar{\eta }$ (constant vaccination).

Thus, when there is no seasonality in the transmission rate, it is optimal to vaccinate at a constant rate throughout the year.

**Fig. 5:**
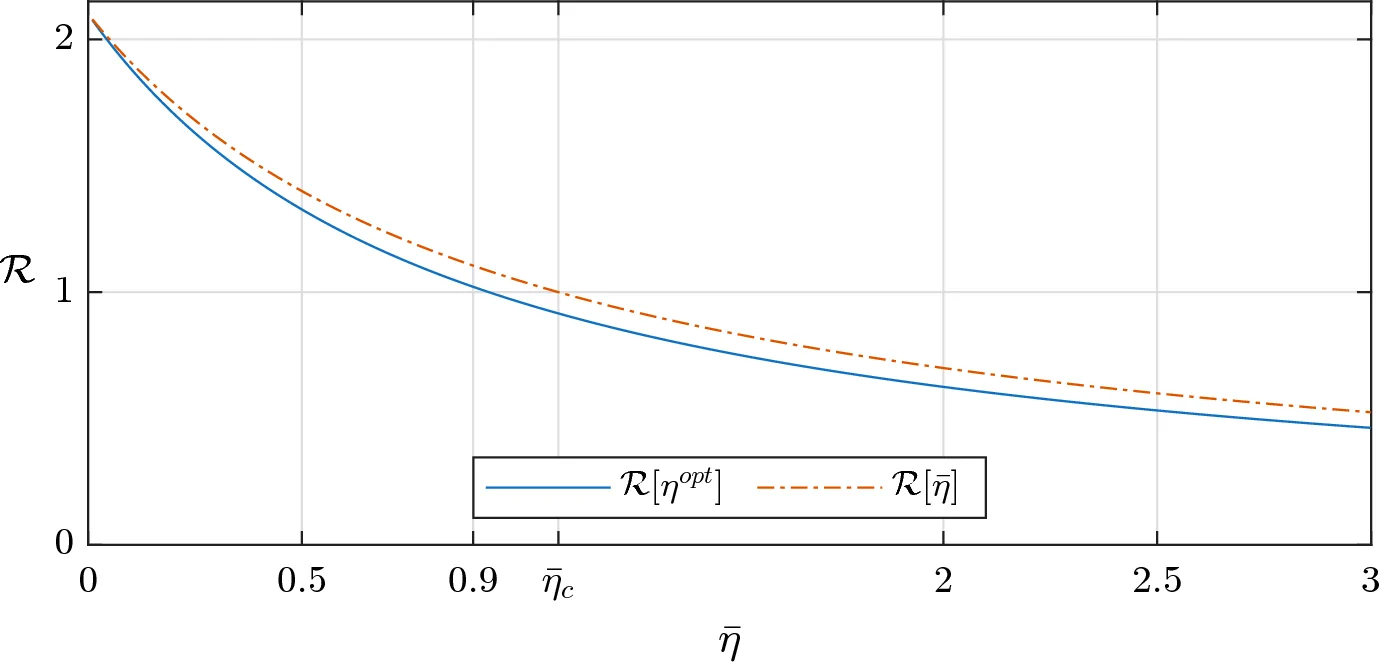
Reproduction numbers $\mathcal {R}[\eta ^\textrm{opt}]$ (solid blue curve) and $\mathcal {R}[\eta ^\textrm{u}]$ (red dash-dotted curve) as a function of $\bar{\eta }$ for the parameters used in Fig. 2

Recall that Problem 1 was motivated by the desire to eliminate a disease (achieve $\mathcal {R}[\eta ]<1$) with a minimal mean vaccination rate $\bar{\eta }$ - see Sect. 2.1. We now address this problem by considering the function $\mathcal {R}^\textrm{min}(\bar{\eta })$ defined by (8), that is reproduction number $\mathcal {R}[\eta ^\textrm{opt}]$ as a function of $\bar{\eta }$, plotted in Fig. 5. The value $\bar{\eta }^\textrm{min}$ for which $\mathcal {R}^\textrm{min}(\bar{\eta }^\textrm{min})=1$ is the *minimal* mean vaccination rate $\bar{\eta }^\textrm{min}$ that can achieve elimination. In the example presented in Fig. 5, $\bar{\eta }^\textrm{min}\approx 0.9$. We super-impose a graph of  $\mathcal {R}[\eta ^\textrm{u}]$ corresponding to a constant profile $\eta ^\textrm{u}(t)=\bar{\eta }$, and observe that, as expected, elimination with a uniform vaccination profile is achieved when $\bar{\eta }\ge \bar{\eta }_c=1.1$, see (7). Thus in this example the yearly amount of vaccines required for elimination is 18% lower when using the optimal vaccination profile, relative to uniform vaccination.

### Monotonicity of the Optimal Vaccination Profile in Dependence on $\bar{\eta }$ - An Open Question

An observation supported by all our numerical results is that when the mean vaccination rate $\bar{\eta }$ increases, the optimal vaccination profile increases at every point, as can be observed in Figs. 2, 3, 4. These observations motivate the following *monotonicity conjecture*:

#### Conjecture 8

Under the assumption of Theorem 4, if $\bar{\eta }_1<\bar{\eta }_2$ then the corresponding optimal profiles satisfy $\eta ^\textrm{opt}_1(t)\le \eta ^\textrm{opt}_2(t)$.

In particular, this implies $\Omega (\eta ^\textrm{opt}_1)\subset \Omega (\eta ^\textrm{opt}_2)$. This conjecture means that increasing the total supply of vaccines will never make it advisable to decrease the vaccination rate at some time of the year. Although this might seem intuitive and is in line with the results about the limit $\bar{\eta }\rightarrow 0+$ and about large $\bar{\eta }$ presented in this section, the validity of this conjecture is not obvious, and we have not been able to prove it. We leave this as an open question for future work.

## Dependence of the Optimal Vaccination Profile on the Rate of Vaccine Decay

We now consider the effect of the rate $\delta _V$ at which vaccine immunity wanes on the optimal vaccination profile. Recall that we set $\delta =\delta _V+\mu$, so that varying the rate of immunity decay is equivalent to varying δ. It is instructive to first consider an example in the limit of scarce vaccines, $\bar{\eta }\rightarrow 0$. In Sect. 4.1, we have shown that the 1–periodic transmission profile (18) with peak transmission at $\left. t=1/2\right.$ gave rise to an optimal vaccination profile concentrated at a point $t^*$ given by (21). Expression (21) implies that $t^*(\delta )$ is monotone increasing with

$$ \lim _{\delta \rightarrow 0+} t^*(\delta )=\frac{1}{4},\qquad \lim _{\delta \rightarrow \infty }t^*(\delta )=\frac{1}{2}, $$

so that, as one may expect, faster waning rate leads to vaccination closer to the transmission peak at t=1/2.

**Fig. 6:**
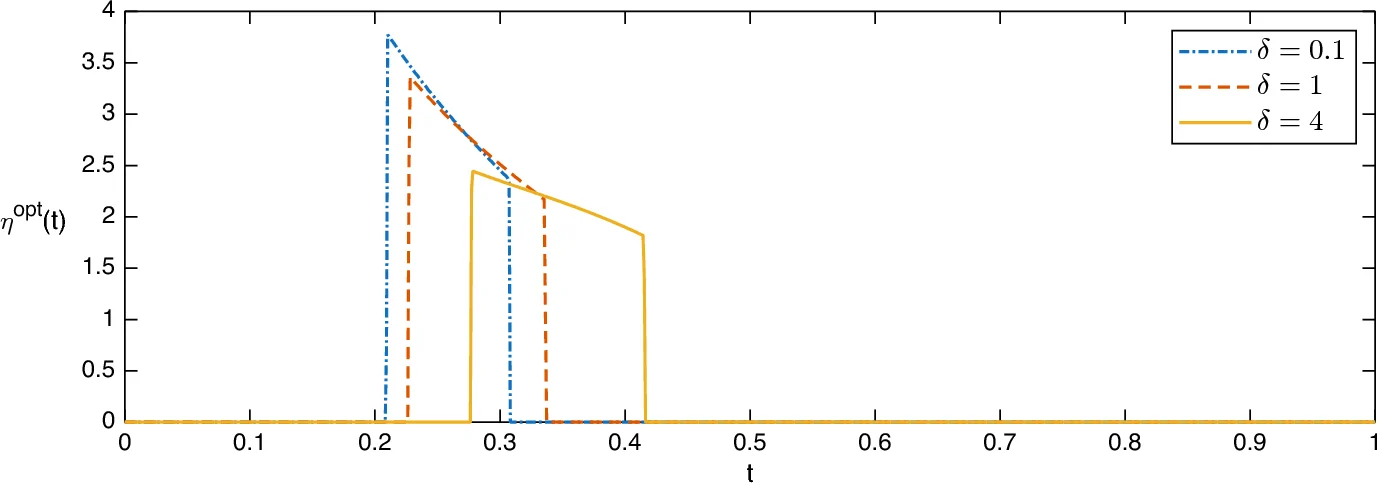
Optimal vaccination profile $\eta ^\textrm{opt}$ for the same seasonality as in Fig. 2 with $\bar{\eta }=1$ and for different values of δ: δ=0.1 (blue dash-dotted curve), δ=1 (red dashed curve), δ=4 (yellow solid curve)

Moving beyond the limit of scarce vaccines, we compare the case considered in Fig. 2 with $\bar{\eta }=1$, for which vaccine immunity lasts on average approximately one year (δ=1), with two extreme cases of long ten-year immunity, δ=0.1 and short three-month immunity, δ=4 (since we assume $\mu =\frac{1}{80}$ we have that $\delta _V\approx \delta$).

We observe that for the different values of δ, the optimal vaccination profiles are qualitatively similar and include a single vaccination interval that is shifted in time and slightly extended in duration as δ increases; see Fig. 6.

## Quantifying the Relative Efficacy of the Optimal Vaccination Profile

Theorem 6 fully characterizes the solution of Problem 1 when a sufficiently large number of vaccines is administered per year. Using this explicit expression, we can quantify the relative reduction in $\mathcal {R}$ when the optimal profile is used, compared to the case in which vaccinations are administered at a constant rate throughout the year, that is the uniform profile $\eta ^\textrm{u}(t)\doteq \bar{\eta }$ is used. Since

$$\begin{aligned} \mathcal {R}[\eta ^\textrm{u}]=\frac{1}{\gamma +\mu }\cdot \frac{\delta }{\bar{\eta }+\delta }\frac{1}{T}\int _0^T \beta (t)dt, \end{aligned}$$

and using (24), we obtain

### Proposition 9

Under the assumptions of Theorem 4, and assuming $\bar{\eta }>\bar{\eta }_m$, we have

25 $$\begin{aligned} \varphi \doteq \frac{\mathcal {R}[\eta ^\textrm{opt}]}{\mathcal {R}[\eta ^\textrm{u}]}=\frac{\left( \int _0^T\sqrt{\beta (t)}dt\right) ^2}{\int _0^T\beta (t)dt}. \end{aligned}$$

Note that Jensen’s inequality implies that, unless β(t) is constant, the ratio φ is strictly smaller than 1. Thus, the uniform profile is optimal if and only if β(t) is constant. Note also the implication that, when $\bar{\eta }>\bar{\eta }_m$, the ratio in (25) depends only on the transmission profile β(t) and, in particular, is independent of $\bar{\eta }$ and δ.

The quantity 1-φ is the relative reduction in $\mathcal {R}$ achieved by the optimal vaccination profile compared the uniform one.

**Fig. 7:**
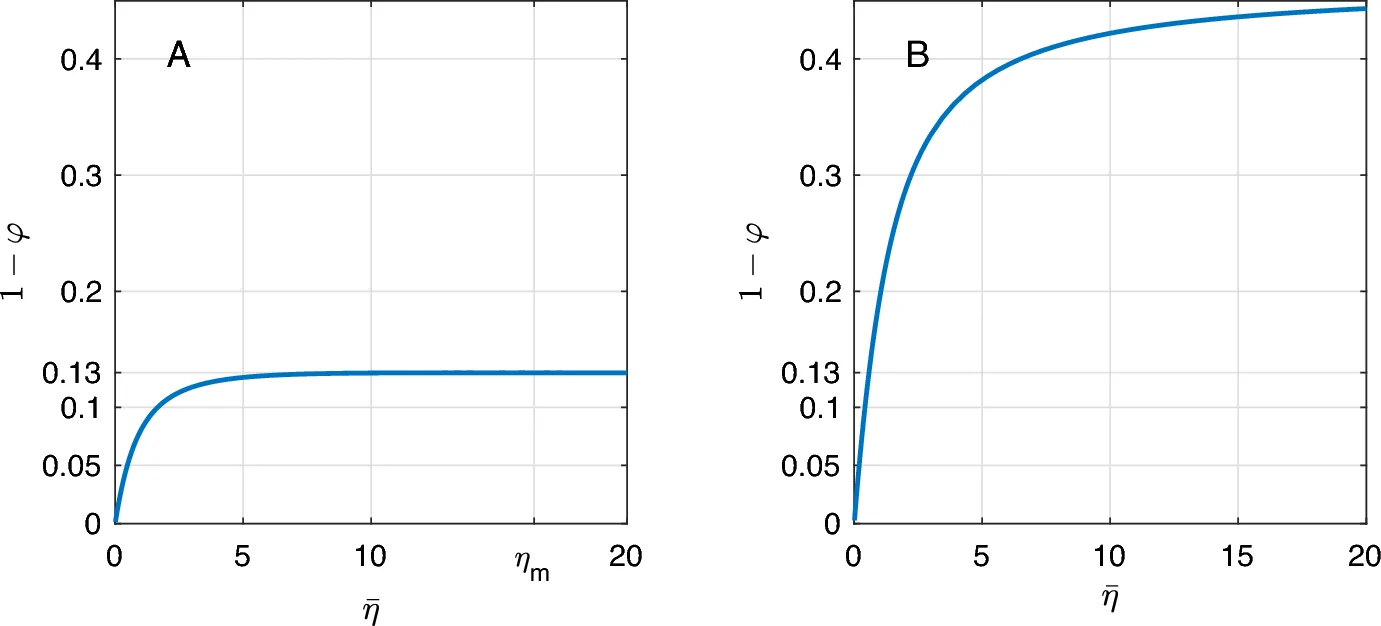
Relative improvement 1-φ where φ is given by (25) as a function of $\bar{\eta }$ for (**A**) The parameters used in Fig. 2. (**B**) Same as A, but with the transmission function (26)

While this reduction in $\mathcal {R}$ can be calculated analytically for $\bar{\eta }>\bar{\eta }_m$ using Proposition 9, for smaller vaccination efforts, $\bar{\eta }<\bar{\eta }_m$, we rely on numerical calculations to obtain φ. In Fig. 7A we graph the relative reduction in $\mathcal {R}$ when the transmission profile is given by (18), with a=0.9, calculated as a function of the average vaccination rate $\bar{\eta }$. When $\bar{\eta }=0$ (no vaccination) we have $\mathcal {R}[\eta ^\textrm{opt}]=\mathcal {R}[\eta ^\textrm{u}]=\frac{\bar{\beta }}{\gamma +\mu }$, so that the relative reduction is 0. We observe numerically (though we have not proven this analytically) that the relative reduction increases monotonically with $\bar{\eta }$. For $\bar{\eta }>\bar{\eta }_m$, in agreement with Proposition 9, we have that φ does not depend on $\bar{\eta }$ and its value 1-ϕ=0.1298 is given by (25).

In the example above, for values of $\bar{\eta }\le 0.5$, the relative improvement is less than 5%. That is, in the realistic scenario that less than 50% of the population is vaccinated annually, the optimal temporal allocation of vaccines improves $\mathcal {R}$ by few percent relative to a uniform allocation of vaccines over time. In the following, we provide an example in which a much larger improvement in $\mathcal {R}$ is obtained. However, note that the dynamics of disease spread is sensitive to changes in $\mathcal {R}$, and thus even a small improvement can have a significant impact. We will consider the impact of using the optimal vaccination profile on the solution of the SIRVS model (1) in Sect. 8.

**Fig. 8:**
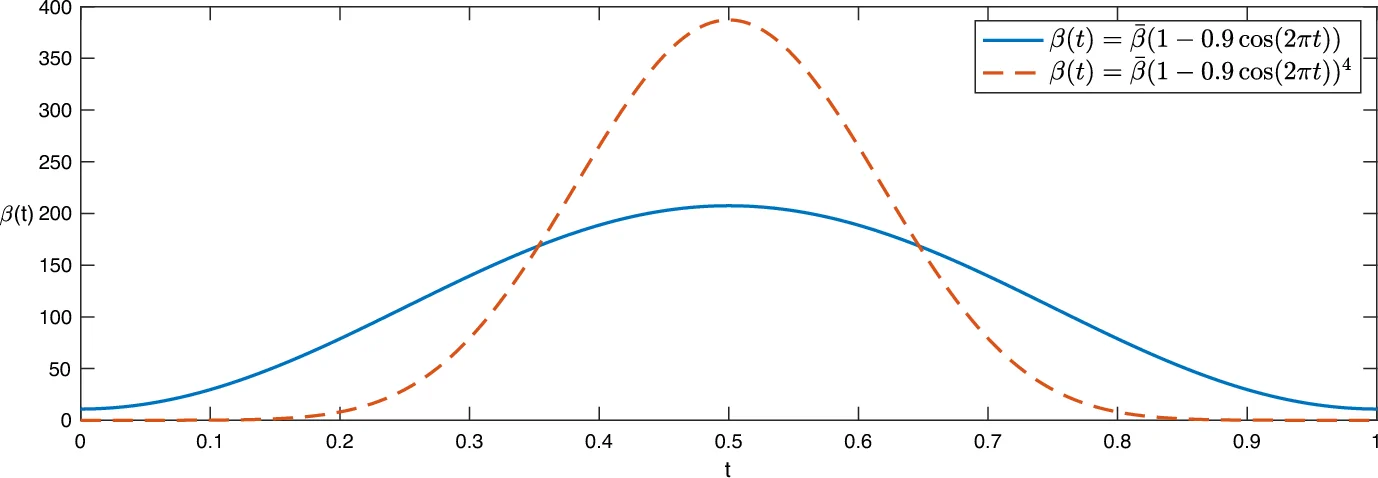
Transmission profiles (18) (blue solid curve) and (26) (red dashed curve)

An arbitrarily high improvement of the optimal reproductive number relative to that obtained by constant-rate vaccination can be achieved when the transmission profile β(t) is concentrated in a short season. To demonstrate this, we compute the relative improvement, 1-φ, for the transmission profile

26 $$\begin{aligned} \beta (t)=\bar{\beta }\cdot \frac{(1-a\cos (2\pi t))^4}{\int _0^1 (1-a\cos (2\pi s))^4\,ds} ,\quad \bar{\beta }=(\gamma +\mu ) {\mathcal {R}}_0. \end{aligned}$$

This profile is more concentrated than the transmission profile (18) used in Fig. 7A, see Fig. 8. As expected, the relative improvement increases sharply with $\bar{\eta }$ and exceeds 40% for large values of $\bar{\eta }$, see Fig. 7B.

## Discontinuous Transmission Rates

In the results presented above, we have assumed that the seasonality profile β(t) is an absolutely continuous function, in which case Theorem 4 shows that the optimal vaccination profile is an absolutely continuous measure. We now address the case in which the function β(t) can have jump discontinuities. This case is relevant since many studies assume piecewise constant seasonality, with jump discontinuities, representing school terms and holiday periods (Buonomo et al. 2018; Stone et al. 2007), in which contact rates among schoolchildren are reduced.

In the following, we will show that this extension entails new phenomena: The optimal vaccination profile, $m^\textrm{opt}$, becomes a mixed measure, which can include both an absolutely continuous component (corresponding to a continuous vaccination rate) and atomic components (pulses of vaccination on specific dates). Note that such components of Dirac δ-function type correspond to points $t^*$ at which the cumulative effort function $\alpha ^\textrm{opt}(t)$ has a jump discontinuity, with the mass of the δ-function, that is, the number of vaccines administered at time $t=t^*$, relative to the total population, given by the jump size $\alpha ^\textrm{opt}(t^*+)-\alpha ^\textrm{opt}(t^*-)$.

We now present a general result concerning the case of a discontinuous seasonality profile, and subsequently we illustrate it by numerical computations and discuss its implications. The following result follows from Gavish and Katriel (Theorem 5, 2026).

### Theorem 10

Assume the *T*-periodic function β(t) satisfies the following:

i) The right and left-hand limits $\beta (t\pm )$ exist at every point *t*, and $\left. \beta (t\pm )>0\right.$.
ii) Denoting by $D_\pm$ the set of discontinuity points of β(t) with an upward and downward jump respectively,
  $$ D_+=\left\{ t\in [0,T) \,| \beta (t+)> \beta (t-)\right\} ,\quad D_-=\left\{ t\in [0,T)\,| \beta (t+)< \beta (t-)\right\} , $$
  and $D=D_+\cup D_-$, the set *D* is finite.
iii) β(t) is differentiable at all points apart from a countable set, and absolutely continuous on every closed subinterval $[a,b]\subset [0,T)\setminus D$.

Then,

1. For $t_1<t_2$ with $[t_1,t_2]\subset [0,T)\setminus D$, we have
  $$\begin{aligned} m^\textrm{opt}([t_1,t_2))=\int _{t_1}^{t_2} \eta ^\textrm{opt}(t)dt, \end{aligned}$$
  with $\eta ^\textrm{opt}$ given by (15),(16), for some value λ.
2. If $t^*\in D_-$ then $t^*\not \in \Omega (m^\textrm{opt})$, i.e.,  $m^\textrm{opt}((t^*-\epsilon ,t^*+\epsilon ))=0$ for ϵ sufficiently small.
3. If $t^*\in D_+$ and if the right and left-hand limits of $\eta ^\textrm{opt}$ at $t^*$ exist and satisfy
  27 $$\begin{aligned} \eta ^\textrm{opt}(t^*-)>0 \;\;{\text{ and } } \eta ^\textrm{opt}(t^*+)>0 ,\end{aligned}$$
  then the optimal vaccination profile $m^\textrm{opt}$ has a Dirac δ-function component located at $t^*$, with mass given by
  28 $$\begin{aligned} \alpha ^\textrm{opt}(t^*+)-\alpha ^\textrm{opt}(t^*-)=\frac{1}{2}\log \left( \frac{\beta (t^*+)}{\beta (t^*-)}\right) . \end{aligned}$$
  At such points the function $S^\textrm{opt}(t)$ has a downward jump with
  $$\begin{aligned} \frac{S(t^*+)}{S(t^*-)}=\sqrt{\frac{\beta (t^*-)}{\beta (t^*+)}}. \end{aligned}$$

Theorem 10 distinguishes between upward and downward jump points of the transmission profile β(t), which lead to different behavior of the optimal vaccination profile. Specifically, a downward jump in the transmission rate at time $t^*$ creates a ‘no-vaccination zone’ in the optimal schedule around that time. At an upward jump of point $t^*$ of β(t), there is a possibility that a vaccination pulse will be given at the point of time $t^*$ at which this jump occurs. However, we have only proved that a pulse occurs under the additional condition (27). The theorem does not characterize the behavior of the optimal vaccination profile at upward jump points of β(t) for which (27) does not hold, and in this sense the theorem is incomplete. The numerical example below will allow us to explore situations not covered by the theorem.

**Fig. 9:**
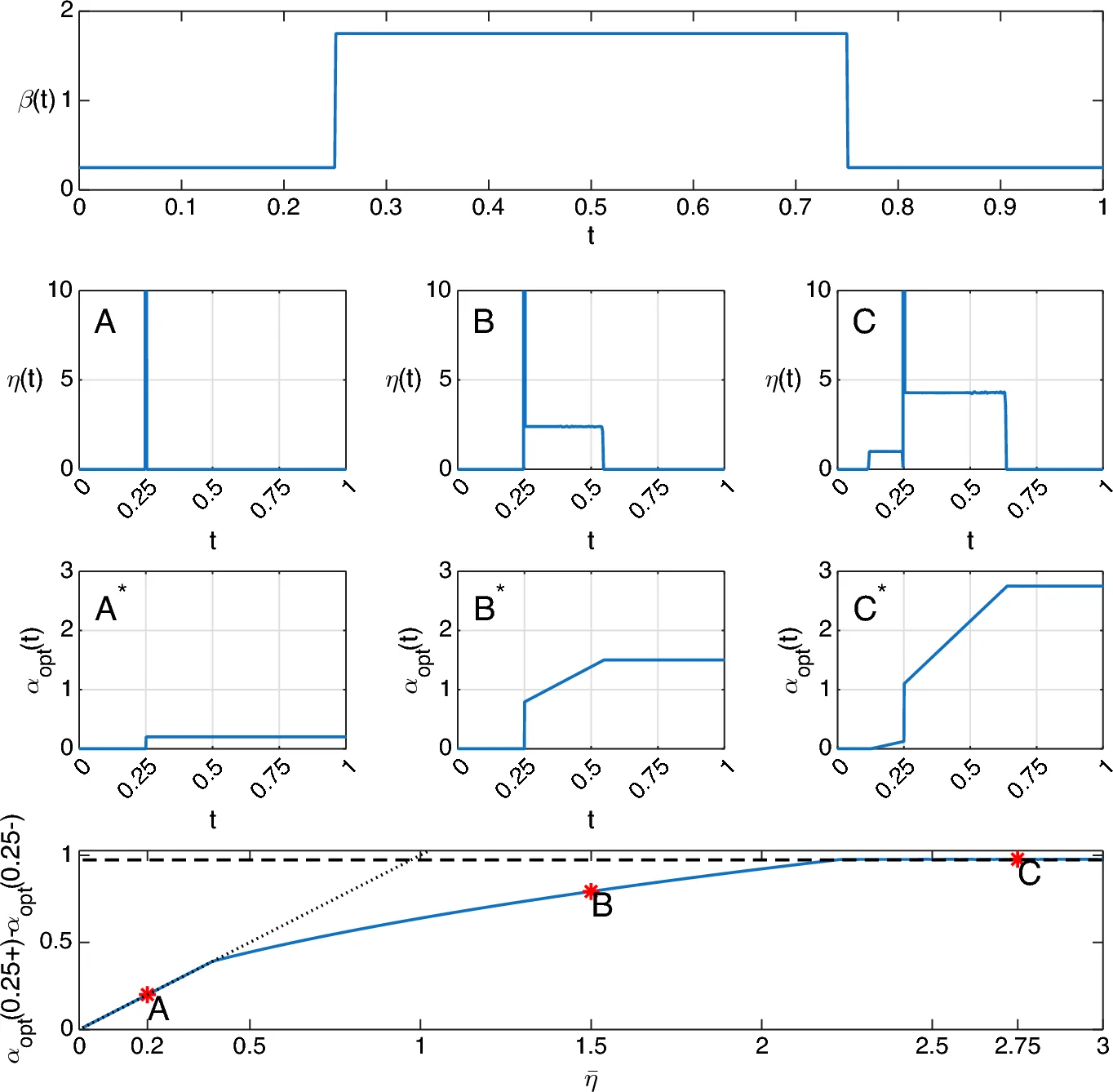
Solution η(t) of Problem 1 for β(t) given by (29), see top graph, δ=1 and different values of $\bar{\eta }$: **A** $\bar{\eta }=0.2$, **B** $\bar{\eta }=1.5$ and **C** $\bar{\eta }=2.75$. The integral $\alpha ^\textrm{opt}(t)=\int _0^t \eta (s)ds$ is presented in graphs $A^*$, $B^*$ and $C^*$, respectively. Bottom graph presented the jump value $\alpha ^\textrm{opt}(0.25+)-\alpha ^\textrm{opt}(0.25-)$ as a function of $\bar{\eta }$

In Fig. 9 we choose the seasonal transmission function which is the 1-periodic extension of

29 $$\begin{aligned} \beta (t)={\left\{ \begin{array}{ll} 1.75 & \frac{1}{4}<t<\frac{3}{4},\\ 0.25 & 0\le t\le \frac{1}{4} \text{ or } \frac{3}{4}\le t<1, \end{array}\right. } \end{aligned}$$

so that transmission is low for half of the year and high for the other half. Here $D_+=\{0.25\}$ and $D_-=\{0.75\}$. We set δ=1, and numerically solve the optimization problem for several values of $\bar{\eta }$. According to conclusion 2 of Theorem 10, we see that it is always the case that $\eta ^\textrm{opt}(t)=0$ for *t* in a neighborhood of $t^*=0.75\in D_-$. Consider now $t^*=0.25\in D_+$, at which β(t) has a upward jump discontinuity. In Fig. 9C we observe that for $\bar{\eta }=2.8$ we have $\eta ^\textrm{opt}(0.25-),\eta ^\textrm{opt}(0.25+)\ne 0$. Therefore, condition (27) is satisfied and, in accordance with conclusion 3 of the theorem, we observe a pulse component of $m^\textrm{opt}$ at $t^*=0.25$, with a jump size in $\alpha ^\textrm{opt}$ as specified by formula (28), see Fig. 9C and the bottom panel of Fig. 9. For $\bar{\eta }=1.5$, we observe that $\eta ^\textrm{opt}(0.25-)=0$, hence Condition (27) does not hold - see Fig. 9B. Therefore, Theorem 10 does not ensure that $m^\textrm{opt}$ has an atomic component at t=0.25. However, we observe (see the bottom graph of Fig. 9) that for $0.4<\bar{\eta }<2.25$, $m^\textrm{opt}$ has a pulse component at t=0.25 with a mass of $\alpha ^\textrm{opt}(0.25+)-\alpha ^\textrm{opt}(0.25-)$ which is monotone increasing with $\bar{\eta }$, and is lower than the value given in (28). We do not have an analytic expression for the mass of the δ-function in this case. Finally, for $\bar{\eta }<0.4$, we observe that the optimal measure $m^\textrm{opt}$ is a pure pulse, consisting of Dirac δ functions with mass $\bar{\eta }$ located at $t^*=0.25$. In this case, we clearly have $\alpha ^{\textrm{opt}}(t^*+)-\alpha ^{\textrm{opt}}(t^*+)=T\bar{\eta }$ since the entire yearly stock of vaccines is administered at this time. Thus, in the case of an upward jump and a sufficiently low vaccination effort, pulse vaccination given at the time of transition from low to high transmission, is the optimal strategy.

Concluding this discussion, we note the gaps that remain between what we have been able to prove analytically and the phenomena observed in the numerical computations. Theorem 10 proves the existence of a pulse component at $t^*\in D_+$ only under the additional assumption (27), but the numerical example shows that a pulse component can exist at $t^*\in D_+$ even when $\eta ^\textrm{opt}(t^*-)=0$. Obtaining necessary and sufficient conditions for existence of a pulse at $t^*\in D_+$, as well as characterizing the mass of the pulse in the case that (27) does not hold, remain interesting questions for further research.

## Solution of the full SIRVS Model for the Optimal Vaccination Profile

The vaccination profile $\eta ^\textrm{opt}$ is chosen so that it minimizes the basic reproduction number associated with the SIRVS model (1). Thus, the problem studied here is most relevant in the case that the amount of available vaccine is sufficient to achieve elimination, that is, when $\mathcal {R}[m^\textrm{opt}]< 1$. If this is not the case, the disease will remain endemic despite vaccination. In particular, the component *S*(*t*) of the solution of the SIRVS model (1) will be different from $S^\textrm{opt}(t)$, which corresponds to the disease-free state, since susceptibles will also be depleted by infections. Indeed, it is not guaranteed that the solution of the SIRVS model (1) will be *T*-periodic even if both β(t) and η(t), are *T*-periodic, as this model can also display subharmonic oscillations and chaotic behavior. If elimination of the disease cannot be achieved, it is of interest to examine to what extent the choice of vaccination profile $m^\textrm{opt}$, which minimizes the reproductive number, improves upon a constant rate vaccination in terms of disease burden. We now explore this through a numerical simulation of the full SIRVS model (1).

**Fig. 10:**
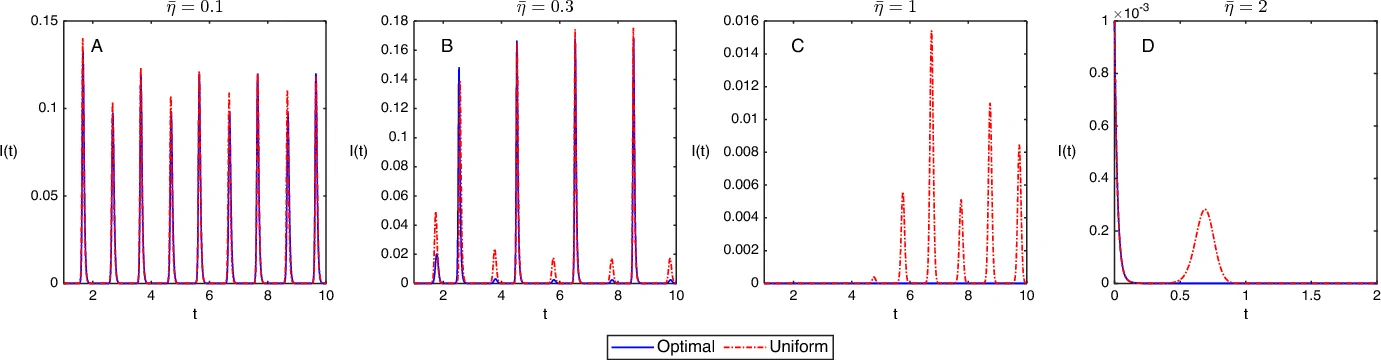
Solution of SIRVS model (1) for $\beta (t)=2.1(\gamma +\mu )(1-0.9\cos (2\pi t))$, γ=52, μ=1/80 and $\delta =\delta _R=1$ with $\eta (t)=\eta ^\textrm{opt}(\bar{\eta })$ for different values of $\bar{\eta }$. **A** $\bar{\eta }=0.1$, **B** $\bar{\eta }=0.3$, **C** $\bar{\eta }=1$ and **D** $\bar{\eta }=2$

**Fig. 11:**
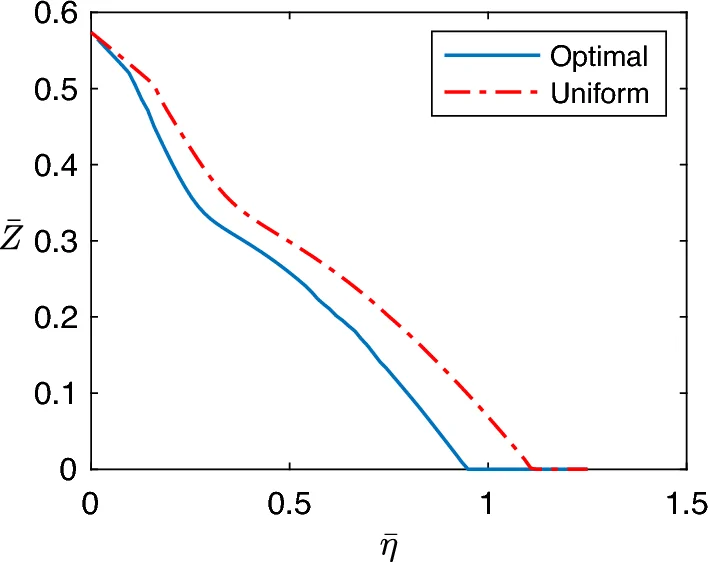
Mean attack rate $\bar{Z}$ (30) as a function of $\bar{\eta }$ for the solution of the SIRVS model (1) with the same parameters as in Fig. 2: $\beta (t)=(\gamma +\mu )\cdot 2.1(1-0.9\cos (2\pi t))$, γ=52, $\mu =2.5\cdot 10^{-4}$ and $\delta =\delta _R=1$

Figure 2 presents optimal vaccination profiles for different values of the average vaccination rate $\bar{\eta }$. Figure 10 explores the prevalence of infection *I*(*t*) in the case of uniform and optimal vaccination rates, based on the SIRVS model (1), using the parameters of Fig. 2. In Fig. 10A, with $\bar{\eta } = 0.1$, the basic reproduction number for uniform vaccination, $\mathcal {R}[\eta ^\textrm{u}] \approx 1.91$, is only slightly higher than $\mathcal {R}[\eta ^\textrm{opt}] \approx 1.88$ for the optimal vaccination profile. This minor difference has a small effect on the long-term dynamics of the corresponding SIRVS solutions; see Fig. 10A. Note that in this case, the SIRVS solution is subharmonic with period 2 for both uniform and optimized vaccination profiles. When $\bar{\eta } = 0.3$, the optimal strategy reduces the basic reproduction number by $\sim 3.8\%$, from $\mathcal {R}[\eta ^\textrm{u}] \approx 1.62$ to $\mathcal {R}[\eta ^\textrm{opt}] \approx 1.56$, reducing the average incidence over time, especially every other year; see Fig. 10B. With $\bar{\eta }=1$, $\mathcal {R}[\eta ^\textrm{opt}]\approx 0.96$ falls below one, ensuring elimination of the disease when the optimal profile is applied, but $R[\eta _u]\approx 1.05$ so that uniform vaccination does not eliminate the disease; see Fig. 10C. Finally, Fig. 10D shows an instance where both $\mathcal {R}[\eta ^\textrm{opt}]$ and $\mathcal {R}[\eta ^\textrm{u}]$ are less than 1, leading to elimination, with the optimal profile achieving a faster reduction in prevalence. Figure 11 complements the numerical study of this example by presenting the mean attack rate (number of cases per year)

30 $$\begin{aligned} \bar{Z}=\gamma \lim _{t\rightarrow \infty }\frac{1}{t}\int _{0}^{t} I(s)ds, \end{aligned}$$

computed numerically, as a function of $\bar{\eta }$. We observe that the mean attack rate decreases with $\bar{\eta }$ and, furthermore, that the optimal vaccination profile results in a lower attack rate than a uniform vaccination, despite the fact that our optimal profile is not specifically designed to minimize the attack rate, but rather the reproductive number.

## Discussion

In this work, we formulated and addressed the problem of optimal periodic vaccination for an epidemiological model with seasonal transmission and waning immunity. We aimed for long-term disease elimination, which leads to the problem of finding a periodic vaccination strategy that minimizes the reproductive number. The resulting formulation presents a rich mathematical problem. Notably, our approach avoids *a priori* assumptions about the regularity of the optimal vaccination profile, prompting us to frame the optimization over a space of measures. This reveals that optimal profiles may include pulses components, highlighting the necessity of our general formulation. Our framework thus goes beyond standard optimal control theory, which typically assumes bounded control functions. Our work (Gavish and Katriel 2026) provided us with the analytical basis for this study.

Our results show that the optimal vaccination profile exists and that, under the appropriate regularity assumptions on the seasonality function β(t), the optimal solution, which is *a priori* an arbitrary measure, is, in fact, absolutely continuous. Moreover, we were able to characterize the functional form of the solution up to the composition of the intervals corresponding to vaccination and non-vaccination periods.

In the case of a discontinuous transmission profile, we showed that the upward and downward jumps in the transmission profile β(t) are associated with a vaccination pulse or a duration without vaccination, respectively. We therefore learn that ‘pulse vaccination’ emerges as the optimal strategy under specific conditions. In practical terms, implementing a perfect vaccination pulse is impossible, but these results provide some guidance for public health campaigns. They suggest that, in relevant cases, concentrating vaccination efforts over a very short period prior to the high-transmission season can be significantly more effective than spreading the same resources over a longer period. Mathematically, this is justified by the fact that the functional $\mathcal {R}[\eta ]$, defined on the space ${\mathcal {M}}$, is continuous with respect to the weak topology on this space (Gavish and Katriel 2026, Lemma 2), which implies that approximating a Dirac δ measure by a pulse-like function will lead to a nearby value of $\mathcal {R}$. While our numerical results are indeed computed using a discretized problem, relying solely on a discrete-time formulation carries a significant drawback: explicit analytical expressions for the optimal profile, such as those we derived in the continuous-time case, are generally unattainable in a discrete formulation. Therefore, despite the mathematical technicalities involved, the continuous-time formulation ultimately yields more transparent and satisfying analytical insights. These continuous, theoretically ideal vaccination profiles can then be used to justify and guide the search for practical, effective interventions among a smaller class of simple, discretized profiles.

In this work we have made the assumption that vaccines are randomly administered to all non-infected individuals. As discussed in Sect. 2, we consider this approach is particularly appropriate in the context of repeated vaccination and waning immunity, as opposed to targeting only the susceptible class (*S*). The latter case, which is appropriate in the case of lifelong immunity, was investigated by Onyango and Müller (2014). A comparison of the nature of the optimal vaccination profile minimizing the reproductive number $\mathcal {R}$ in the two cases shows major differences. For example, characterization of the optimal vaccination profile in Theorem 2 of Onyango and Müller (2014) shows that when the vaccine availability ($\bar{\eta }$ in our notation) is sufficiently low, the optimal vaccination profile is pure pulse vaccination of all susceptible individuals at times $t_0+kT$ ($k\in \mathbb {Z}$), where $t_0$ is defined as the maximum point of an explicitly given function defined in terms of the transmission profile β. This is in sharp contrast with our result here, which shows that, in the case that β satisfies the conditions of Theorem 4, then when $\bar{\eta }$ is sufficiently large then the vaccination profile is everywhere positive, with density given explicitly by (22), so that vaccination is carried out throughout the year. For higher values of $\bar{\eta }$, the optimal profile in Onyango and Müller (2014) involves intervals without vaccination and intervals in which *S* is brought down to zero and maintained at this value by continuous vaccination. This qualitative behavior differs significantly from the optimal profiles derived here, underscoring that the underlying assumptions regarding the mode of vaccination are highly significant for policy design.

While the assumption that all non-infected individuals are vaccinated is more appropriate in the case of repeated vaccination than the assumption that only susceptibles are vaccinated, it remains an oversimplification, and to achieve a more realistic description we should take into account a refractory period following vaccination during which an individual is not eligible for re-vaccination. This will have a double-edged effect: it will improve efficiency by reducing the redundancy involved in vaccinating individuals who are currently protected, but, on the negative side, some individuals will lose protection before becoming eligible for re-vaccination, thus increasing *S*, and hence $\mathcal {R}$. It is possible to model this more realistic scenario by dividing each of the classes *V* and *S* into two classes - those not eligible for re-vaccination and those eligible, with transitions among them. It will be of interest, but mathematically more difficult, to study Problem 1 for such a model.

Future work should also incorporate greater epidemiological realism along other dimensions into the models, and seek to understand the impact of such variations on optimal vaccination profiles. Regarding the course of infection, one should consider the SEIR model or models including a more general infectious period distribution Krylova and Earn (2013). Such extensions are highly non-trivial, since for such models there is usually no explicit expression for the quantity $\mathcal {R}$ in the case of periodic forcing. In such cases, the basic reproduction number must be defined as the spectral radius of an associated integral operator (Bacaër 2007; Bacaër and Ait Dads 2012; Nakata and Kuniya 2010), raising additional technical challenges in treating the optimization problem.

The dynamics of decay of immunity, which was modelled here in the simplest possible way, in which a vaccinated person remains protected for a random exponentially-distributed duration, can be modelled with other residence-time distributions as well as accounting for partial protection as immunity wanes (Barbarossa and Rost 2015). In such cases, the simple equation (4) will be replaced by an equation with distributed delays, which will, in general, not be explicitly solvable. Since our analysis exploited the explicit solution (5), solving the Problem 1 for such models will require new approaches. We expect that explicit expressions for optimal vaccination profiles, such as those obtained in Theorems 4,6, will often not be attainable for more general models.

Another direction in which investigations should be extended involves other natural criteria for optimality of a vaccination strategy. The current study focuses on the elimination of the disease and therefore considers the minimization of the reproduction number $\mathcal {R}$ (6). However, achieving elimination may not always be feasible, in which case the focus could be on minimizing the number of infections averaged over time in the endemic state. In models with seasonality, we do not expect that the vaccination profile which minimizes the reproductive number, $\mathcal {R}$, will also minimize the total number of infections (Bacaër and Gomes 2009). The problem of minimizing the number of infections is a difficult one in view of the fact that the endemic steady state may be periodic (but for which an explicit expression is not available) or chaotic, and multi-stability may occur, so that different behaviors are possible even for the same parameters and vaccination profile (Earn et al. 2000; Barrientos et al. 2017; Brugnago et al. 2023). For example, in Fig. 10 the model inputs are 1-periodic, but the endemic steady state is 2-periodic. Although the numerical results show that the optimal vaccination profile designed to minimize $\mathcal {R}$ also reduce the cumulative number of infections (Fig. 11), a profile designed specifically to minimize infections would perform even better in this regard.

We conclude that optimzing time-dependent vaccination in the case of seasonally dependent transmission rates involves interesting modelling issues and mathematical questions, and it is hoped that the progress made in this work on one version of this problem will inspire further developments. We note, finally, that the problem of optimized resource utilization in periodic settings arises in many other applications, such as drug administration (Cortés-Ríos et al. 2022; Ghanaatpishe and Fathy 2017; Ghanaatpishe et al. 2017; Varigonda et al. 2008), chemotherapy (Belfo and Lemos 2021; Swierniak and Ploskonski 2010), chronotherapy (Adam 2019), harvesting (Jong et al. 2024; Tang et al. 2006; Xiao et al. 2006; Braverman and Mamdani 2008), pest control (Cardoso et al. 2009; Tang et al. 2010), etc. Therefore the ideas and methodologies presented herein and in Gavish and Katriel (2026) should be relevant to future work in additional domains of theoretical biology.

## Data Availability

The Matlab codes used to produce the graphs presented in the manuscript are available at https://github.com/NGavish/Optimal-vaccination-in-seasonal-epidemics.git
